# Procedural robotic surgery training: a UK pan-specialty trainee Delphi consensus study

**DOI:** 10.1007/s11701-025-02582-0

**Published:** 2025-08-21

**Authors:** Matthew Harris, Aidan Bannon, Justin W. Collins

**Affiliations:** 1 Association of Surgeons in Training, Royal College of Surgeons of England, 38-43 Lincoln’s Inn Fields, London, WC2A 3PE UK; 2 Robotic and Digital Surgery Trainee’s Committee, Royal College of Surgeons of England, 38-43 Lincoln’s Inn Fields, London, UK; 3 Division of Surgery and Interventional Science, University College London, London, UK; 4 Division of Cancer Sciences, University of Manchester, Manchester, UK

**Keywords:** Robotic surgery, training, education, credentialing, curriculum

## Abstract

Robotic surgery has become increasingly prevalent in the UK, with 65% of the population living within a 30-min drive of a robotic surgical centre. However, many surgical trainees lack access to structured robotic training, highlighting the need for standardised pathways to equip future surgeons. This study seeks to determine a consensus amongst UK surgical trainees on the opinion of essential components of procedural robotic training. A trainee-led Delphi consensus study was conducted with 85 surgical trainees representing the spectrum of surgical specialism and training grades. A steering group consisting of a surgical expert and trainee representatives developed and refined statements across key themes, including robotic curricula, credentialling structure, assessment standards, error metrics and access to training. Trainee feedback was collected through three iterative survey rounds, with consensus defined as ≥ 80% agreement or disagreement. Consensus was achieved for 82 of 141 statements. Trainees strongly supported the integration of robotic training into surgical curricula, emphasising the importance of metrics-based assessment and credentialling in device, basic and procedural training. Key recommendations included platform-agnostic training, benchmarking, proficiency-based progression, and video-based assessments for procedural skills. Additional endorsements included revalidation every 5 years and centralised registries for robotic cases. Flexible access to training, beginning at higher specialty levels, was also advocated. This study establishes a robust consensus amongst UK surgical trainees, highlighting the acceptability of credentialling in robotic surgery. The findings provide a framework for developing equitable and standardised robotic training pathways to ensure the preparedness of future surgeons in an evolving surgical landscape.

**Supplementary Information:**

The online version contains supplementary material available at 10.1007/s11701-025-02582-0.

## Introduction

There has been steadily growing adoption of robotic surgery into standard UK surgical practice in recent years with a global trend demonstrating increasing replacement of laparoscopic and open surgery services with robotic technology [1, 2]. Robotic surgery has become increasingly accessible and visible to patients in the UK, with 65% of the population living within a 30-min drive of a robotic surgical centre [3].

General surgery, gynaecology and urology procedures represent the majority of robotic procedures performed worldwide [4]. The global robotic surgery market is anticipated to continue to grow, expanding to $19 billion by 2027 [5]. A recent review of current and emerging minimally invasive soft tissue robotic platforms identified 23 single or multiport platforms at different stages of development and evaluation, highlighting a rapidly changing surgical landscape [6]. Investment in multiple product launches and promotion will further drive adoption. For example, over 1 million robotic hip and knee procedures have been performed using Stryker’s Mako robotic navigation system [7].

Surgical training to date has, not kept pace with this rapid expansion. There is increasing recognition of the need to train robotic surgeons in established training pathways, but many UK trainees currently do not have access to a robot. Moreover, trainees consistently report that robotic surgery will be both relevant and important to their future surgical careers [8–10]. With the recent notable exception of gynaecology, current specialty training curricula do not refer to the role of robotic surgery in UK surgical training [11, 12]. A recent national survey of surgeons in training found that 95% of trainees from all specialties concluded that robotic surgery would be integral to their future service delivery, it is therefore likely that access to training will be an important aspect for recruitment and retainment of surgical staff within the NHS. This same survey reported a 98% agreement that a standardised pre-procedural robotic core curriculum (PPCRC) would be beneficial to training and would potentially lead to more equitable access for trainees [13]. This consensus suggested that a universal PPCRC should be integrated into surgical training, focussing on e-learning, device training and simulation with defined and validated assessment standards that should enable progression to procedural based training.

Credentialling is defined by the UK General Medical Council as a process that formally accredits a doctor in attaining competence in a ‘discrete area of practice’. There have been calls for surgeon credentialling in the standardised delivery of robotic training to ensure both operative and patient safety [14]. In response to the need for standardisation of robotic training, recommendations have been developed in the US to define benchmarks and criteria to ensure technical proficiency and to maintain accreditation as a robotic surgeon [15]. There is also increasing awareness of the need for trainees to be objectively assessed in both surgical technique and device training [16]. Recent guidelines published by the Royal College of Surgeons in England have suggested minimum standards to determine robotic proficiency including attainment of basic robotic skills, minimum number of cases performed and regular review of operative metrics and performance [17].

This pan-specialty study seeks to determine whether UK surgical trainees support a national robotic surgery credentialling framework and the components which they deem essential in the development of future robotic training programmes.

## Methodology

### Evidence acquisition

A steering group was formed with representation from the Association of Surgeons in Training (AB), the Robotic and Digital Surgery Training Committee (MH), and a senior robotic surgeon with expertise in consensus methodology (JC). This steering group reviewed and discussed existing evidence, which was previously collated during the consensus development of a pre-procedural core robotic curriculum by Burke et al. [13]. The quality of existing evidence has previously been described as low to moderate, with the majority of evidence comprising consensus statements, expert opinion or small qualitative studies. Evidence published following Burke et al. [13] was reviewed systematically and incorporated into the Delphi statements where needed.

In total, 61 statements were devised, consisting of 142 individual items. These were identified within five themes:

1. The need for a robotic curriculum
2. Structure of robotic credentialling
3. Assessment in robotic credentialling
4. Assessment of errors and metrics
5. Access to credentialling

### Participant selection

The Association of Surgeons in Training (ASiT) is an independent professional body focussed on promoting the highest standards in surgical training. It represents surgical trainees at all levels, from all surgical specialties. ASiT has representation on the council of four Royal Colleges of Surgeons, Specialist Advisory Committees and the Joint Committee on Surgical Training (JCST). We invited contributions from trainee representatives from each of the member organisations within our Council. The invited participants represented the breadth of surgical practice in the UK with the aim of developing a pan-specialty, pan-grade consensus. The training organisations represented are presented in Supplementary Table 3. Invited participants were all elected members of UK trainee surgical societies.

### Delphi process

Delphi methodology was subsequently used as a method to gain trainee consensus to enable generation of a list of recommendations. The principle features of this process ensure anonymity, and repeated iterations of questionnaires with specific feedback to concentrate findings towards a group response [18].

Before trainee participation, the steering group produced a short informative video summarising the current robotic landscape including context and definitions of credentialling and objective metric-based assessment for trainees prior to participation. A link to the video has been made available, *Supplementary Evidence 1*.

Prior to participation in the Delphi exercise, written information was supplied within each questionnaire to clarify the format and structure of the consensus process.

**Fig. 1:**
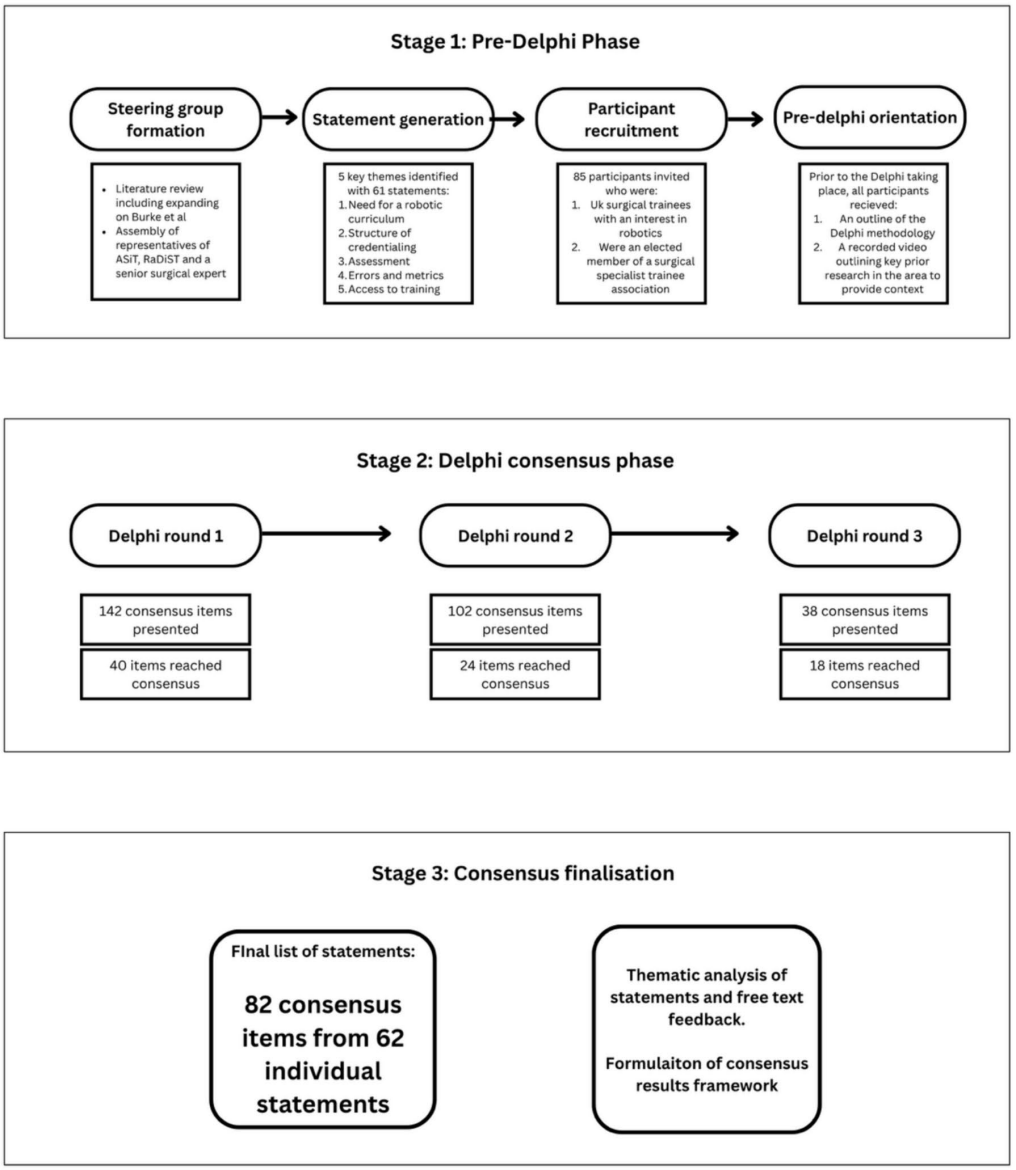
Delphi consensus methodology

A three-round Delphi process using an internet survey (Google Forms, Mountain View, CA) was undertaken over a 27-day period. A complete list of the survey items have been made available, Supplementary information 1. Participants were asked to indicate their level of ‘agreement’ or ‘disagreement’. Proposed statements and criteria were generated by the steering group, with all responses anonymised. Consensus was defined as 80% agreement or disagreement of responses in each round. The initial survey was analysed and statements with > 80% agreement or disagreement were removed from subsequent rounds. Repeated iterations of anonymous voting continued over three rounds, where an individual’s vote in the next round was informed by knowledge of the entire group’s results in the previous round. Those statements which did not reach 80% agreement by the third round were not included in the final list of credentialling criteria and were, therefore, excluded from the final recommendations. In the Delphi process, the finding of consensus was more important than the level of consensus, with the full consensus process outlined in Figure 1.

## Results

In total, 85 trainees participated in the Delphi process. There was a 95% (81/85) response rate from participants in the final round, with 100% (85/85) and 91% (77/85) in the first and second rounds, respectively.

### Demographics

Of the 85 participants, the majority were represented by higher specialty surgical trainees (n = 56, 66%). There was representation from every surgical specialty and at every stage of training from medical student to post-CCT fellow. Colorectal (n = 15, 17.6%), upper GI (n = 11, 12.9%), and urology trainees (n = 10, 11.8%) were the predominant specialty groups. Training grades and specialty groups are outlined in Table 1 and Table 2, respectively.

**Table 1:** Training grades represented in the consensus exercise

Grade	Number (n = 85)	%
Medical student	4	4.7%
FY1	2	2.4%
FY2	2	2.4%
CST1/CST2	12	14.1%
Junior clinical fellow	1	1.2%
ST3-ST5	30	35.3%
ST6-ST8	26	30.6%
Post CCT fellow	2	2.4%
Senior clinical fellow	6	7.1%

**Table 2:** Specialty groups represented in the consensus exercise

Specialty	Number (n = 85)	%
Colorectal	15	17.6%
Upper GI	11	12.9%
HPB	8	9.4%
Transplant	7	8.2%
Vascular	3	3.5%
Orthopaedics	7	8.2%
Paediatric surgery	3	3.5%
Plastic surgery	8	9.4%
Cardiothoracic	4	4.7%
Emergency general surgery	1	1.2%
Urology	10	11.8%
Neurosurgery	1	1.2%
ENT	3	3.5%
Maxillofacial	4	4.7%

Following conclusion of the 3 rounds of the Delphi process, 82 of 141 items achieved consensus (> 80% agreement or disagreement). There was a 91.8% agreement that there should be credentialling in robotic surgery, with 96% of trainees agreeing that robotic training should be standardised.

Table 3 outlines the statements achieving consensus within the common themes and level of agreement/disagreement with each statement.

**Table 3:**
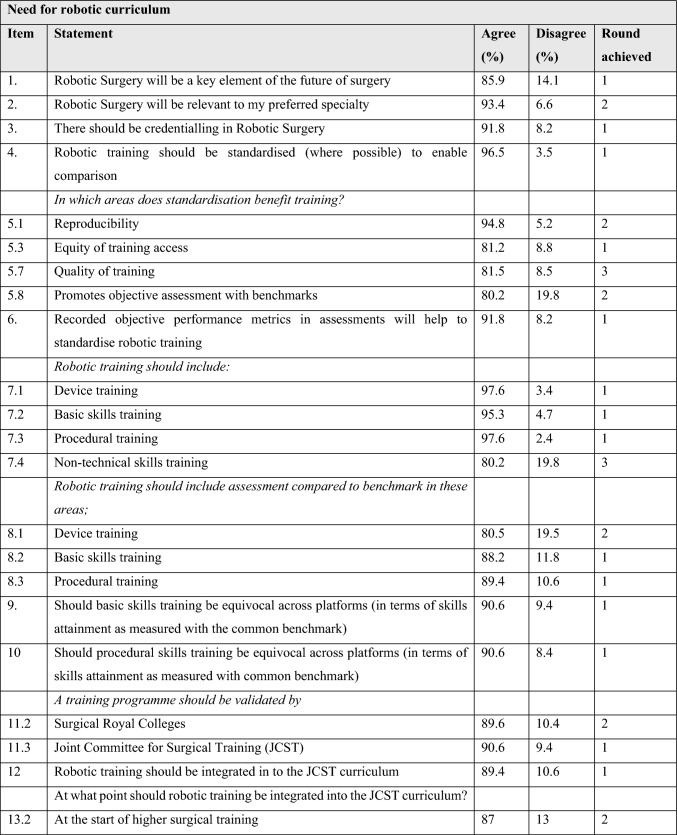

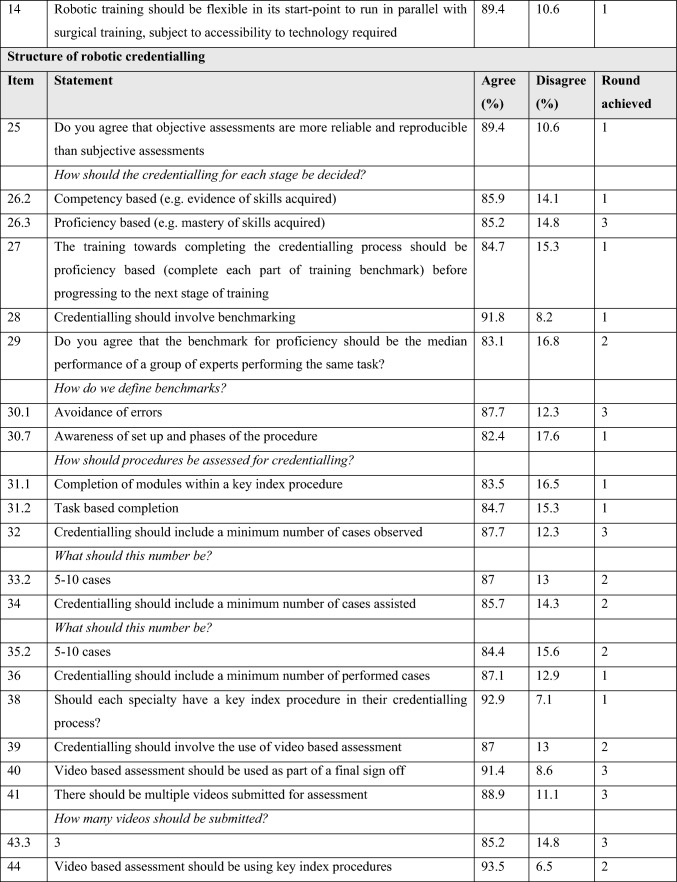

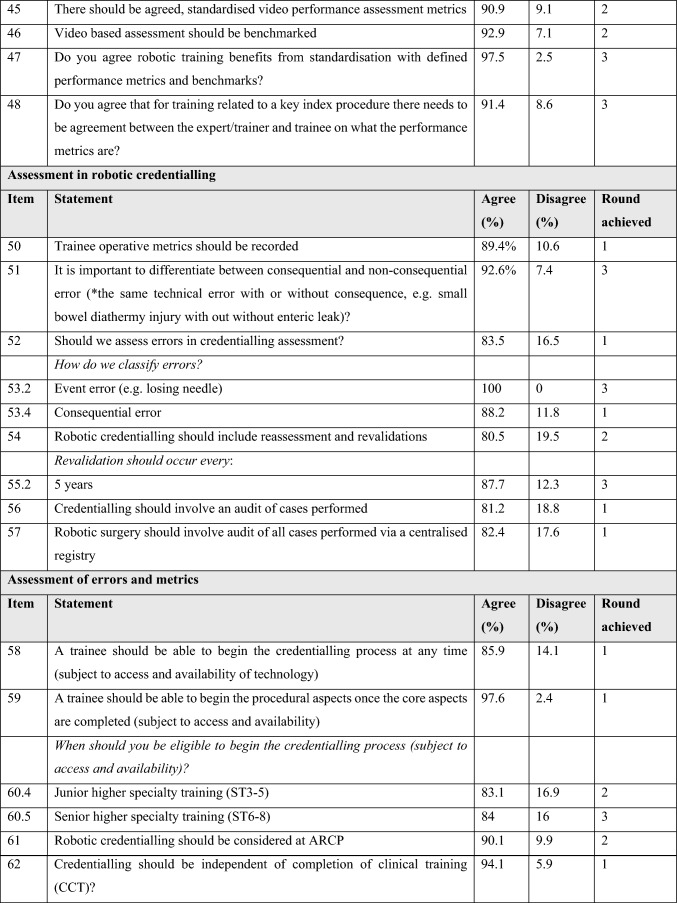
Statements reaching consensus

### Need for a curriculum

There was strong consensus that credentialling is acceptable to UK trainees and that robotic training should be integrated into the UK surgical curriculum at a suitable point. There was strong consensus that recording of objective performance metrics would help to standardise training. Trainees strongly agreed that basic and procedural skills attainment should be benchmarked and be able to be assessed across different robotic training platforms. When asked at what point robotic procedural training should be integrated into the curriculum, the consensus was that this should begin at the start of higher surgical training (ST3-5), but that this should be flexible in its delivery, and subject to accessibility to the technology required. There was agreement that procedural training programmes should be validated by the Royal Colleges of Surgery and the JCST in favour of NHS or industry bodies.

### Structure of credentialling

There was strong consensus that a robotic curriculum should include device training, basic skills training, simulation training and supervised procedural training, and these should be credentialed to optimise feedback and evaluation. It is recognised that device training is delivered by industry to promote safe implementation. Procedural training should be validated and development overseen by societies, with access to key opinion leaders and the current evidence. Whilst there was consensus that non-technical skills training should be included in a robotic curriculum, this did not reach threshold for consensus.

Trainees agreed that credentialling in procedural skills training should include multiple training environments including wet lab, cadaveric and high-fidelity non-cadaveric model training, emergency open conversion courses and that there should be a modular approach to procedural training. Trainees also agreed that basic skills training should be standardised irrespective of specialty but that procedural training should be procedure specific and final sign off should include use of a specialty specific (index) procedure. In addition, trainees agreed that videos of optimised technique, simulation training and mentorship/preceptorship would support and enhance training during the credentialling process.

### Assessment

Trainees strongly agreed that objective assessments are more reliable and reproducible than subjective assessments. There was consensus for both competency and proficiency-based assessment, but with a stronger consensus for the former. Trainees did agree with the principle of benchmarking and that proficiency represents the median performance of a group of experts performing the same task or procedure. Whilst there was less consistent agreement on which benchmarks to measure, avoidance of errors and awareness of set up and agreement on the phases of procedures were considered important elements that reached consensus. Trainees reached consensus that the minimum number of cases observed and assisted should be between 5–10, respectively. There was strong consensus that a total number of cases performed should be considered for accreditation but there was no agreement as to how this should be defined. Trainees agreed that credentialling should involve the use of video-based assessment and that these should be used for index procedures using objective assessment metrics. Trainees strongly agreed that these metrics should be defined and mutually agreed in advance with their trainers.

### Errors and metrics

There was a strong consensus that defined and agreed objective metrics should be recorded during training and that errors should be considered in the credentialling process. There was agreement that consequential and non-consequential errors should be differentiated. There was agreement that ‘event’ and ‘consequential’ errors should be classified but there was disagreement when pre-errors or non-consequential errors were considered.

Trainees agreed that credentialling should include revalidation at 5-year intervals. Finally, there was consensus that an audit of cases should be performed during the credentialling process and that all robotic surgery should be included in a centralised registry.

### Access to credentialling

There was consensus that trainees should be able to begin the credentialling process at any time, commencing with the device training and basic skills training elements, but that trainees should not be eligible to access procedural training until higher specialty training (ST3 +). There was strong agreement that a trainee should be able to progress with procedural aspects of training upon completion of a basic skills training programme. Whilst there was strong agreement that robotic credentialling should be considered at ARCP, the majority of trainees agreed that credentialling should be independent of the completion of clinical training certification.

## Discussion

We used Delphi methodology to demonstrate consensus amongst surgical trainees in the United Kingdom on the acceptability of credentialling in robotic training. Trainees agreed that formal credentialling processes in robotic surgical training would improve objectivity, reproducibility and quality assurance, whilst promoting equity of access to robotic training. This consensus study builds on previously published work on trainees’ views of the requirements of a PPCRC and that credentialling should include device, basic skills training and simulation and patient-based procedural training [13]. A structured and standardised robotic curriculum is anticipated to substantially benefit surgical trainees by providing clarity, equitable access, and improved proficiency through benchmarked metrics. For patients, enhanced trainee proficiency may translate into better surgical outcomes and reduced complication rates. Furthermore, implementing accessible and standardised robotic training may positively influence workforce retention by addressing trainees’ expectations for modern and technology-driven surgical careers, thereby improving recruitment, job satisfaction and career longevity within the NHS.

Trainees agreed that robotic training should be standardised using objective performance metrics with benchmarks, which is in keeping with the evolving culture of metric-based assessment in surgical training as recommended in the UK [12, 19]. Trainees demonstrated a willingness to embrace new technologies such as video-based assessment and support the use of registries to audit robotic outcomes. The emergence of computer vision deep learning technology that can detect errors in technical skills is likely to set a foundation for future assessment of proficiency in robotic skills training in this regard [20]. Boal et al. objectively evaluated a core robotic skills training curriculum for junior trainee surgeons using proficiency-based metrics demonstrating excellent inter-rater reliability and establishment of a benchmark [21]. Despite a 60% pass rate, all participants rated this highly and stated they would recommend the course. Proficiency-based progression may be a cornerstone of robotic training, but there is currently limited application of this in surgical training programmes and a national strategy will be required in the future implementation of a metrics-based standardised robotic curriculum with defined benchmarks. Future robotic curricula development will also need to ensure that novel training technologies incorporated into the curricula reflect the same learning objectives to enable a continuum of learning [22].

A unique strength of this study is the pan-specialty, pan-grade representation of surgical trainees, demonstrating the diversity of surgical training in the UK. Over 60% of trainees who participated were registered in higher specialty trainee programmes; however, there was still representation from more junior trainees. The attrition rate was low across consecutive rounds with > 90% participation across the three rounds. Participants strongly agreed that basic skills training and procedural skills training should be equivalent across platforms (in terms of skills attainment against a common benchmark). Multi-platform robotic training is an emerging area of interest in the context of simulation and metrics-based progression. The SIMULATE trial demonstrated that junior surgeons who underwent additional simulation training had overall higher metrics-based scores and required less time to achieve operative proficiency [23]. Larkins et al. suggested that console skills are transferable between platforms in a multi-platform cross-over simulation exercise and demonstrated equivalent safety metrics across platforms [24]. Future credentialling processes and robotic training will need to take the growing market into account and will rely on agreed benchmarks in metrics-based assessments to guarantee safety and equity in training and accreditation.

This study aims to establish the surgical trainee as an important stakeholder in future discussions on robotic training; however, we recognise the limitations of presenting a single consensus opinion. Whilst the consensus clearly identifies essential curriculum components, significant challenges remain for implementation within the UK medical education system. Resource constraints, variability in access to robotic platforms, and potential resistance to adopting additional credentialling processes could hinder rapid integration. In addition, aligning robotic training requirements with existing competencies outlined by the Joint Committee on Surgical Training (JCST) and Surgical Royal Colleges may require substantial curriculum redesign and increased collaboration between regulatory bodies, industry partners and training institutions. Specialty specific guidelines have been developed to help guide the safe and reproducible delivery of robotic training programmes but these are yet to translate and be applied universally [25–27]. Similar consensus studies conducted internationally, including the European Robotic Surgery Consensus [27] and initiatives by the International Medical Robotics Academy [25], highlight comparable core components such as platform-agnostic training, proficiency benchmarks and structured credentialling. However, differences remain, particularly regarding benchmarks for procedural training and methods for revalidation, underscoring the need for a unified international standard adaptable to UK surgical curricula [28].

**Fig. 2:**
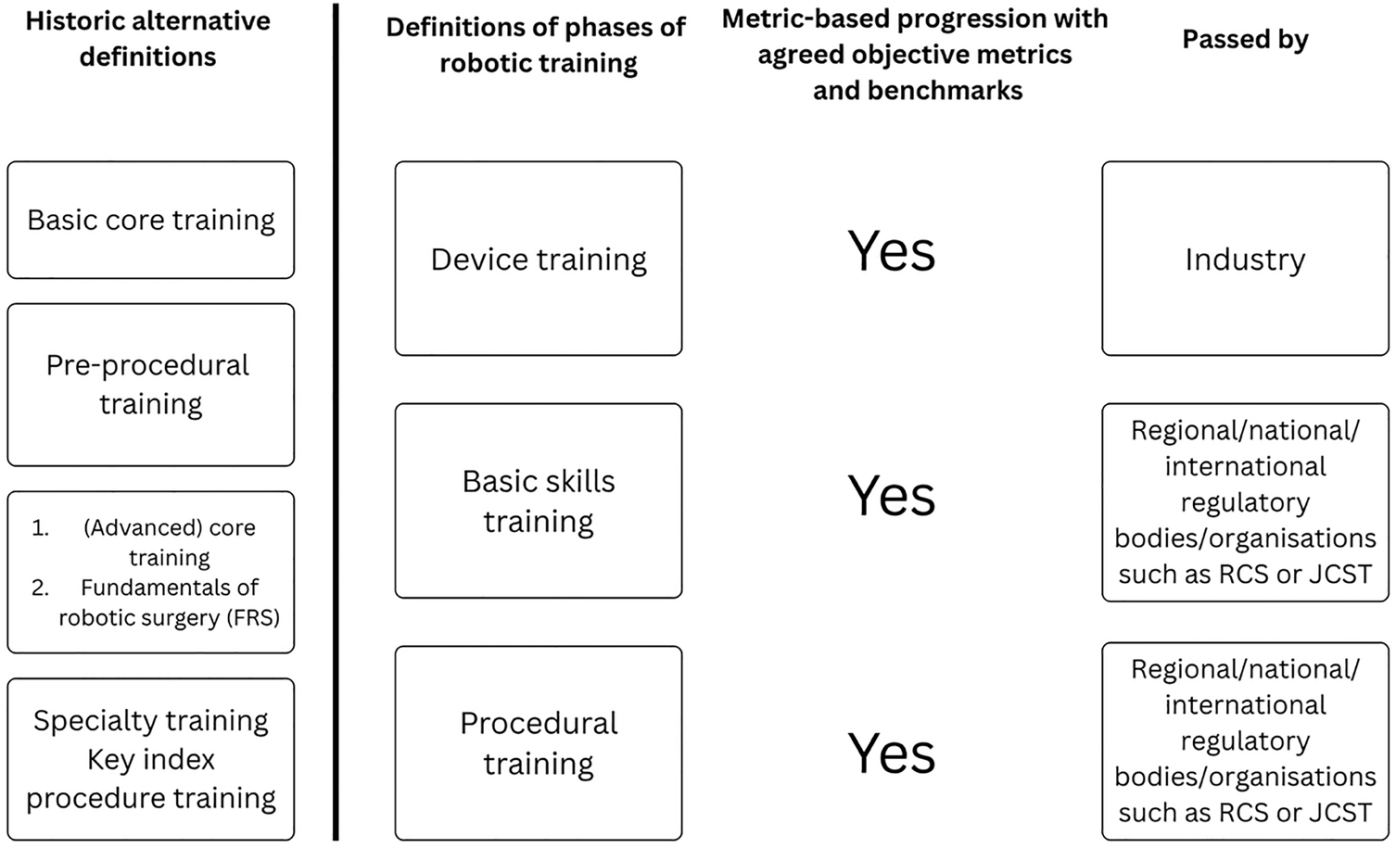
Definitions of phases of robotic training with suggested ‘sign offs’ at each phase

Many of the consensus statements in our study within the domains related to ‘structure’ and ‘assessment’ have the potential to enrich the training experience for UK trainees and could lead to a future passport with a record of signoff for each phase of robotic training, namely device training, basic skills training and also specify the completed procedural training, as outlined in Fig. 2. We suggest the definition of the phases of training in this table to prevent future ambiguity and enable standardisation across modern curricula. Trainee stakeholder participation and engagement may mitigate perceived barriers to technology adoption and accelerate adoption of video-based assessment in robotic training. Most of the trainees participating in this study are naïve to robotic training but have a unique insight into the challenges of training and therefore are well equipped to help guide implementation as innovation continues to fill the space [29]. We understand that this consensus is limited to UK trainees’ experience and may not adequately reflect the experiences of trainees universally [30]. Nevertheless, we hope the consensus findings outlined here will serve as a foundational framework that can be adapted to guide national and international robotic training needs.

Trainees in this UK study felt strongly that the Royal Colleges and JCST should be responsible for the validation and accreditation of robotic surgery training programmes. The barriers to robotic training in the UK are well versed but the pace of expansion of robotic surgery will only continue to grow and surgical trainees must not be left behind [8–10]. To build on the work from this trainee consensus, we recommend the formation of an expert multispecialty, multistakeholder group to define the essential components of a UK robotic curriculum for trainees. This will benefit from guidance on a platform-agnostic training programme to drive agreement and adoption of metrics-based training. The work presented in this consensus and the PPCRC by Burke et al. highlights that trainees are key stakeholders in this process [13].

In conclusion, this pan-grade, pan-specialty Delphi consensus of UK surgical trainees confirms the acceptability of credentialling in robotic surgery. The use of agreed benchmarked metrics-based training in diverse training environments will enable the standardisation and more equitable delivery of robotic training, whilst enhancing the training experience.

## Supplementary Information

Below is the link to the electronic supplementary material.

Supplementary file1 (DOCX 34 KB)

## Data Availability

The data that support the findings of this study are available upon reasonable request from the corresponding author. Due to the nature of the Delphi methodology and the anonymisation of participant responses, some data may be subject to restrictions to maintain participant confidentiality. Supplementary materials, including the complete list of survey statements, are available as part of the published supplementary information. No datasets were generated or analysed during the current study.
