# Correction: Procedural robotic surgery training: a UK pan-specialty trainee Delphi consensus study

**DOI:** 10.1007/s11701-025-02726-2

**Published:** 2025-09-02

**Authors:** Matthew Harris, Aidan Bannon, Justin W. Collins

**Affiliations:** 1 Association of Surgeons in Training, Royal College of Surgeons of England, 38-43 Lincoln’s Inn Fields, London, WC2A 3PE UK; 2 Robotic and Digital Surgery Trainee’s Committee, Royal College of Surgeons of England, 38-43 Lincoln’s Inn Fields, London, UK; 3 Division of Surgery and Interventional Science, University College London, London, UK; 4 Division of Cancer Sciences, University of Manchester, Manchester, UK

**Correction: Journal of Robotic Surgery (2025) 19:501** **https://doi.org/10.1007/s11701-025-02582-0**

In the original version of this article, in the author group “On behalf of the Association of Surgeons in Training” was missing.

The correct author group are as follows

Matthew Harris, Aidan Bannon, Justin W. Collins, On behalf of the Association of Surgeons in Training

In Table 3 of this article, entries were incorrectly given as

**Table 3** Statements reaching consensus

**Table:** 

Item	Statement	Agree (%)	Disagree (%)	Round achieved
Need for robotic curriculum				
1.	Robotic Surgery will be a key element of the future of surgery	85.9	14.1	1
2.	Robotic Surgery will be relevant to my preferred specialty	93.4	6.6	2
3.	There should be credentialling in Robotic Surgery	91.8	8.2	1
4.	Robotic training should be standardised (where possible) to enable comparison	96.5	3.5	1
	*In which areas does standardisation benefit training?*			
5.1	Reproducibility	94.8	5.2	2
5.3	Equity of training access	81.2	8.8	1
5.7	Quality of training	81.5	8.5	3
5.8	Promotes objective assessment with benchmarks	80.2	19.8	2
6.	Recorded objective performance metrics in assessments will help to standardise robotic training	91.8	8.2	1
	*Robotic training should include:*			
7.1	Device training	97.6	3.4	1
7.2	Basic skills training	95.3	4.7	1
7.3	Procedural training	97.6	2.4	1
7.4	Non-technical skills training	80.2	19.8	3
	*Robotic training should include assessment compared to benchmark in these areas;*			
8.1	Device training	80.5	19.5	2
8.2	Basic skills training	88.2	11.8	1
8.3	Procedural training	89.4	10.6	1
9.	Should basic skills training be equivocal across platforms (in terms of skills attainment as measured with the common benchmark)	90.6	9.4	1
10	Should procedural skills training be equivocal across platforms (in terms of skills attainment as measured with common benchmark)	90.6	8.4	1
	*A training programme should be validated by*			
11.2	Surgical Royal Colleges	89.6	10.4	2
11.3	Joint Committee for Surgical Training (JCST)	90.6	9.4	1
12	Robotic training should be integrated in to the JCST curriculum	89.4	10.6	1
	At what point should robotic training be integrated into the JCST curriculum?			
13.2	At the start of higher surgical training	87	13	2
14	Robotic training should be flexible in its start-point to run in parallel with surgical training, subject to accessibility to technology required	89.4	10.6	1
Structure of robotic credentialling				
25	Do you agree that objective assessments are more reliable and reproducible than subjective assessments	89.4	10.6	1
	*How should the credentialling for each stage be decided?*			
26.2	Competency based (e.g. evidence of skills acquired)	85.9	14.1	1
26.3	Proficiency based (e.g. mastery of skills acquired)	85.2	14.8	3
27	The training towards completing the credentialling process should be proficiency based (complete each part of training benchmark) before progressing to the next stage of training	84.7	15.3	1
28	Credentialling should involve benchmarking	91.8	8.2	1
29	Do you agree that the benchmark for proficiency should be the median performance of a group of experts performing the same task?	83.1	16.8	2
	*How do we define benchmarks?*			
30.1	Avoidance of errors	87.7	12.3	3
30.7	Awareness of set up and phases of the procedure	82.4	17.6	1
	*How should procedures be assessed for credentialling?*			
31.1	Completion of modules within a key index procedure	83.5	16.5	1
31.2	Task based completion	84.7	15.3	1
32	Credentialling should include a minimum number of cases observed	87.7	12.3	3
	*What should this number be?*			
33.2	5-10 cases	87	13	2
34	Credentialling should include a minimum number of cases assisted	85.7	14.3	2
	*What should this number be?*			
35.2	5-10 cases	84.4	15.6	2
36	Credentialling should include a minimum number of performed cases	87.1	12.9	1
38	Should each specialty have a key index procedure in their credentialling process?	92.9	7.1	1
39	Credentialling should involve the use of video based assessment	87	13	2
40	Video based assessment should be used as part of a final sign off	91.4	8.6	3
41	There should be multiple videos submitted for assessment	88.9	11.1	3
	*How many videos should be submitted?*			
43.3	3	85.2	14.8	3
44	Video based assessment should be using key index procedures	93.5	6.5	2
45	There should be agreed, standardised video performance assessment metrics	90.9	9.1	2
46	Video based assessment should be benchmarked	92.9	7.1	2
47	Do you agree robotic training benefits from standardisation with defined performance metrics and benchmarks?	97.5	2.5	3
48	Do you agree that for training related to a key index procedure there needs to be agreement between the expert/trainer and trainee on what the performance metrics are?	91.4	8.6	3
Assessment in robotic credentialling				
50	Trainee operative metrics should be recorded	89.4%	10.6	1
51	It is important to differentiate between consequential and non-consequential error (*the same technical error with or without consequence, e.g. small bowel diathermy injury with out without enteric leak)?	92.6%	7.4	3
52	Should we assess errors in credentialling assessment?	83.5	16.5	1
	*How do we classify errors?*			
53.2	Event error (e.g. losing needle)	100	0	3
53.4	Consequential error	88.2	11.8	1
54	Robotic credentialling should include reassessment and revalidations	80.5	19.5	2
	*Revalidation should occur every*:			
55.2	5 years	87.7	12.3	3
56	Credentialling should involve an audit of cases performed	81.2	18.8	1
57	Robotic surgery should involve audit of all cases performed via a centralised registry	82.4	17.6	1
Assessment of errors and metrics				
58	A trainee should be able to begin the credentialling process at any time (subject to access and availability of technology)	85.9	14.1	1
59	A trainee should be able to begin the procedural aspects once the core aspects are completed (subject to access and availability)	97.6	2.4	1
	*When should you be eligible to begin the credentialling process (subject to access and availability)?*			
60.4	Junior higher specialty training (ST3-5)	83.1	16.9	2
60.5	Senior higher specialty training (ST6-8)	84	16	3
61	Robotic credentialling should be considered at ARCP	90.1	9.9	2
62	Credentialling should be independent of completion of clinical training (CCT)?	94.1	5.9	1

But should have been

**Table 3** Statements reaching consensus

**Table:** 

Need for robotic curriculum				
Item	Statement	Agree (%)	Disagree (%)	Round achieved
1.	Robotic Surgery will be a key element of the future of surgery	85.9	14.1	1
2.	Robotic Surgery will be relevant to my preferred specialty	93.4	6.6	2
3.	There should be credentialling in Robotic Surgery	91.8	8.2	1
4.	Robotic training should be standardised (where possible) to enable comparison	96.5	3.5	1
	*In which areas does standardisation benefit training?*			
5.1	Reproducibility	94.8	5.2	2
5.3	Equity of training access	81.2	8.8	1
5.7	Quality of training	81.5	8.5	3
5.8	Promotes objective assessment with benchmarks	80.2	19.8	2
6.	Recorded objective performance metrics in assessments will help to standardise robotic training	91.8	8.2	1
	*Robotic training should include:*			
7.1	Device training	97.6	3.4	1
7.2	Basic skills training	95.3	4.7	1
7.3	Procedural training	97.6	2.4	1
7.4	Non-technical skills training	80.2	19.8	3
	*Robotic training should include assessment compared to benchmark in these areas*			
8.1	Device training	80.5	19.5	2
8.2	Basic skills training	88.2	11.8	1
8.3	Procedural training	89.4	10.6	1
9.	Should basic skills training be equivocal across platforms (in terms of skills attainment as measured with the common benchmark)	90.6	9.4	1
10	Should procedural skills training be equivocal across platforms (in terms of skills attainment as measured with common benchmark)	90.6	8.4	1
	*A training programme should be validated by*			
11.2	Surgical Royal Colleges	89.6	10.4	2
11.3	Joint Committee for Surgical Training (JCST)	90.6	9.4	1
12	Robotic training should be integrated in to the JCST curriculum	89.4	10.6	1
	At what point should robotic training be integrated into the JCST curriculum?			
13.2	At the start of higher surgical training	87	13	2
14	Robotic training should be flexible in its start-point to run in parallel with surgical training, subject to accessibility to technology required	89.4	10.6	1

Structure of robotic credentialing				
Item	Statement	Agree (%)	Disagree (%)	Round achieved
15	Credentialing is acceptable for UK trainees	92.9	7.1	1
16	Credentialing would promote standardisation of training	92.9	7.1	1
	*Training for credentialing should include the following elements to optimise feedback and evaluation*			
17.1	Device training	91.8	8.2	1
17.2	Basic skills training	90.6	9.4	1
17.3	Simulation training	85.9	14.1	1
17.4	Supervised procedural training	84.7	15.3	1
	*The training towards completing the credentialing process should include various training environments to support and enhance training:*			
18.2	Videos of optimised technique	86.4	13.6	3
18.4	Simulation training	88.2	11.8	1
18.6	Modular approach to training	80	20	1
18.7	Mentorship/preceptorship	83.5	16.5	1
	*Credentialing in basic skills should involve:*			
19.1	Dry lab technical skills training	87.1	12.9	1
19.2	Wet lab technical skills training	81.2	18.8	3
	*Credentialing in procedural skills should involve*			
20.2	Wet lab technical skills training	81.2	18.8	1
20.4	Cadaveric training	81.5	18.5	3
20.5	High-fidelity non cadaveric model training	82.7	17.3	3
20.6	Emergency open conversion course	86.4	13.6	3
21	Should credentialing assessment be standardised for device training irrespective of specialty? (*if assessment is mandatory)	87	13	2
22	Should credentialing assessment be standardised for basic skills training irrespective of specialty? (*if assessment is mandatory)	80	20	1
23	Credentialing related to procedural training should be specialty specific	90.6	9.4	1
24	The final sign off should be using a specialty-specific procedure (key index procedure)	91.8	8.2	1

Assessment in robotic training				
Item	Statement	Agree (%)	Disagree (%)	Round achieved
25	Objective assessments are more reliable and reproducible than subjective assessments	89.4	10.6	1
	*How should the credentialling for each stage be decided?*			
26.2	Competency based (e.g. evidence of skills acquired)	85.9	14.1	1
26.3	Proficiency based (e.g. mastery of skills acquired)	85.2	14.8	3
27	The training towards completing the credentialling process should be proficiency based (complete each part of training benchmark) before progressing to the next stage of training	84.7	15.3	1
28	Credentialling should involve benchmarking	91.8	8.2	1
29	Do you agree that the benchmark for proficiency should be the median performance of a group of experts performing the same task?	83.1	16.8	2
	*How do we define benchmarks?*			
30.1	Avoidance of errors	87.7	12.3	3
30.7	Awareness of set up and phases of the procedure	82.4	17.6	1
	*How should procedures be assessed for credentialling?*			
31.1	Completion of modules within a key index procedure	83.5	16.5	1
31.2	Task based completion	84.7	15.3	1
32	Credentialling should include a minimum number of cases observed	87.7	12.3	3
	*What should this number be?*			
33.2	5-10 cases	87	13	2
34	Credentialling should include a minimum number of cases assisted	85.7	14.3	2
	*What should this number be?*			
35.2	5-10 cases	84.4	15.6	2
36	Credentialling should include a minimum number of performed cases	87.1	12.9	1
38	Should each specialty have a key index procedure in their credentialling process?	92.9	7.1	1
39	Credentialling should involve the use of video based assessment	87	13	2
40	Video based assessment should be used as part of a final sign off	91.4	8.6	3
41	There should be multiple videos submitted for assessment	88.9	11.1	3
	*How many videos should be submitted?*			
43.3	3	85.2	14.8	3
44	Video based assessment should be using key index procedures	93.5	6.5	2
45	There should be agreed, standardised video performance assessment metrics	90.9	9.1	2
46	Video based assessment should be benchmarked	92.9	7.1	2
47	Do you agree robotic training benefits from standardisation with defined performance metrics and benchmarks?	97.5	2.5	3
48	Do you agree that for training related to a key index procedure there needs to be agreement between the expert/trainer and trainee on what the performance metrics are?	91.4	8.6	3

Errors and metrics				
Item	Statement	Agree (%)	Disagree (%)	Round achieved
50	Trainee operative metrics should be recorded	89.4%	10.6	1
51	It is important to differentiate between consequential and non-consequential error (*the same technical error with or without consequence, e.g. small bowel diathermy injury with out without enteric leak)?	92.6%	7.4	3
52	Should we assess errors in credentialling assessment?	83.5	16.5	1
	*How do we classify errors?*			
53.2	Event error (e.g. losing needle)	100	0	3
53.4	Consequential error	88.2	11.8	1
54	Robotic credentialling should include reassessment and revalidations	80.5	19.5	2
	*Revalidation should occur every*:			
55.2	5 years	87.7	12.3	3
56	Credentialling should involve an audit of cases performed	81.2	18.8	1
57	Robotic surgery should involve audit of all cases performed via a centralised registry	82.4	17.6	1

Access to credentialing				
Item	Statement	Agree (%)	Disagree (%)	Round achieved
58	A trainee should be able to begin the credentialling process at any time (subject to access and availability of technology)	85.9	14.1	1
59	A trainee should be able to begin the procedural aspects once the core aspects are completed (subject to access and availability)	97.6	2.4	1
	*When should you be eligible to begin the credentialling process (subject to access and availability)?*			
60.4	Junior higher specialty training (ST3-5)	83.1	16.9	2
60.5	Senior higher specialty training (ST6-8)	84	16	3
61	Robotic credentialling should be considered at ARCP	90.1	9.9	2
62	Credentialling should be independent of completion of clinical training (CCT)?	94.1	5.9	1

