# Supplementary material for: Procedural robotic surgery training: a UK pan-specialty trainee Delphi consensus study
Source: J Robot Surg. 2025 Aug 21;19(1):501. doi: 10.1007/s11701-025-02582-0 (PMC12367954; doi:10.1007/s11701-025-02582-0)
Supplement: Supplementary file 1 — Supplementary file1 (DOCX 34 KB) [file 11701_2025_2582_MOESM1_ESM.docx]

**Supplementary information:**

**Supplementary table 1: Comprehensive list of statements and items with responses**

|  | **Need for a curriculum** | | | |
| --- | --- | --- | --- | --- |
| Item | Statement | Round 1 | Round 2 | Round 3 |
| 1 | Robotic Surgery will be a key element of the future of surgery | **85.9%**  (12.9% unsure) | Reached consensus | Reached consensus |
| 2 | Robotic Surgery will be relevant to my preferred specialty | 75.3%  (18.8% unsure) | **93.4%** | Reached consensus |
| 3 | There should be credentialling in Robotic Surgery | **91.8%** | Reached consensus | Reached consensus |
| 4 | Robotic training should be standardised (where possible) to enable comparison | **96.5%** | Reached consensus | Reached consensus |
|  | *In which areas does standardisation benefit training?* | | | |
| 5.1 | Reproducibility | 72.9% | **94.8%** | Reached consensus |
| 5.2 | Faster learning curve | 65.9% | 41.6% | 65.4% |
| 5.3 | Equity of training access | **81.2%** | Reached consensus | Reached consensus |
| 5.4 | Flexibility in training | 41.2% | 14.3% | 7.4% |
| 5.5 | Equity of assessment | 70.6% | 63.9% | 74.1% |
| 5.6 | Predictability (safer outcomes) | 65.9% | 40.3% | 48.1% |
| 5.7 | Quality of training | 74.1% | 74% | **81.5%** |
| 5.8 | Promotes objective assessment with benchmarks | 75.3% | **80.2%** | Reached consensus |
| 5.9 | Continued improvement in training and assessment | 61.2% | 32.5% | 33.1% |
| 6 | Recorded objective performance metrics in assessments will help to standardise robotic training | **91.8%** | Reached consensus | Reached consensus |
|  | *Robotic training should include:* | | | |
| 7.1 | Device training | **97.6%** | Reached consensus | Reached consensus |
| 7.2 | Basic skills training | **95.3%** | Reached consensus | Reached consensus |
| 7.3 | Procedural training | **97.6%** | Reached consensus | Reached consensus |
| 7.4 | Non-technical skills training | 68.2% | 70.1% | **80.2%** |
|  | *Robotic training should include assessment compared to benchmark in these areas:* | | | |
| 8.1 | Device training | 76.5% | **80.5%** | Reached consensus |
| 8.2 | Basic skills training | **88.2%** | Reached consensus | Reached consensus |
| 8.3 | Procedural training | **89.4%** | Reached consensus | Reached consensus |
| 8.4 | Non-technical skills training | 42.4% | 35.1% | 66.7% |
| 9 | Should basic skills training be equivocal across platforms (in terms of skills attainment as measured with common benchmark) | **90.6%** | Reached consensus | Reached consensus |
| 10 | Should procedural skills training be equivocal across platforms (in terms of skills attainment as measured with common benchmark) | **90.6%** | Reached consensus | Reached consensus |
|  | *A training programme should be validated by (can tick multiple boxes):* | | | |
| 11.1 | GMC | 18.8% | 5.2% | n.a |
| 11.2 | Surgical Royal Colleges | 78.8% | **89.6%** | Reached consensus |
| 11.3 | JCST | **90.6%** | Reached consensus | Reached consensus |
| 11.4 | NHSE | 16.5% | 2.6% | n.a |
| 11.5 | Industry | 10.6% | 2.6% | n.a |
| 12 | Robotic training should be integrated into the JCST curriculum (at a suitable point) | **89.4%** | Reached consensus | Reached consensus |
|  | *At what point should robotic training be integrated into the JCST curriculum?* | | | |
| 13.1 | Start of CST | 21.2% | 16.9% | n.a |
| 13.2 | Start of HST | 64.7% | **87%** | Reached consensus |
| 13.3 | At Subspecialty training | 25.9% | 19.5% | n.a |
| 13.4 | After CCT | 8.2% | 6.5% | n.a |
| 13.5 | As early as possible | 24.7% | 19.5% | n.a |
| 13.6 | It should not be integrated | 5.9% | 1.3% | n.a |
| 14 | Robotic training should be flexible in its start-point to run in parallel with surgical training, subject to accessibility to technology required | **89.4%** | Reached consensus | Reached consensus |

|  | **Credentialling – Structure** | | | |
| --- | --- | --- | --- | --- |
| Item | Statement | Round 1 | Round 2 | Round 3 |
| 15 | Credentialling is acceptable for UK trainees | **92.9%** | Reached consensus | Reached consensus |
| 16 | Credentialling would promote standardisation of training | **92.9%** | Reached consensus | Reached consensus |
|  | *Training for credentialling should include the following elements, to optimise feedback and evaluation:* | | | |
| 17.1 | Device training | **91.8%** | Reached consensus | Reached consensus |
| 17.2 | Basic Skills training | **90.6%** | Reached consensus | Reached consensus |
| 17.3 | Simulation training | **85.9%** | Reached consensus | Reached consensus |
| 17.4 | Supervised procedural training | **84.7%** | Reached consensus | Reached consensus |
| 17.5 | Non technical skills training | 57.6% | 51.9% | 56.8% |
| 17.6 | Telepresence | 49.4% | 20.8% | 29.6% |
|  | *The training towards completing the credentialling process should include various training environments to support and enhance training (tick all that apply)* | | | |
| 18.1 | E-Learning (theory based) | 74.1% | 59.7% | 69.1% |
| 18.2 | Videos of optimised technique | 76.5% | 74% | **86.4%** |
| 18.3 | Baseline assessment | 65.9% | 42.9% | 58% |
| 18.4 | Simulation training | **88.2%** | Reached consensus | Reached consensus |
| 18.5 | Bedside assistance | 60% | 35.1% | 39.5% |
| 18.6 | Modular approach to procedural training | **80%** | Reached consensus | Reached consensus |
| 18.7 | Mentorship/preceptorship | **83.5%** | Reached consensus | Reached consensus |
| 18.8 | Telepresence | 47.1% | 11.7% | 22.2% |
|  | *Credentialling in basic skills training should involve (Tick all that apply)* | | | |
| 19.1 | Dry lab technical skills training | **87.1%** | Reached consensus | Reached consensus |
| 19.2 | Wet lab technical skills training | 76.5% | 77.9% | **82.7%** |
| 19.3 | XR simulation training | 64.7% | 40.3% | 72.8% |
| 19.4 | Cadaveric training | 62.4% | 31.2% | 23.5% |
| 19.5 | High-fidelity non-cadaveric model training | 64.7% | 42.9% | 46.9% |
|  | *Credentialling in procedural skills training should involve (Tick all that apply)* | | | |
| 20.1 | Dry lab technical skills training | 65.9% | 49.4% | 40.7% |
| 20.2 | Wet lab technical skills training | **81.2%** | Reached consensus | Reached consensus |
| 20.3 | XR simulation training | 61.2% | 24.7% | 32.1% |
| 20.4 | Cadaveric training | 79% | 63.2% | **81.5%** |
| 20.5 | High-fidelity non-cadaveric model training | 76.5% | 61% | **82.7%** |
| 20.6 | Emergency open conversion course | 75.3% | 62.3% | **86.4%** |
| 21 | Should credentialling assessment be standardised for device training irrespective of specialty? [If assessment mandatory] | 74.1% | **87%** | Reached consensus |
| 22 | Should credentialling assessment be standardised for basic skills training irrespective of specialty | **80%** | Reached consensus | Reached consensus |
| 23 | Credentialling related to procedural training should be specialty specific | **90.6%** | Reached consensus | Reached consensus |
| 24 | The final sign off should be using a specialty-specific procedure (key index procedure) | **91.8%** | Reached consensus | Reached consensus |

|  | **Assessment** | | | |
| --- | --- | --- | --- | --- |
| **Item** | **Statement** | **Round 1** | **Round 2** | **Round 3** |
| 25 | Do you agree that objective assessments are more reliable and reproducible than subjective assessments? | **89.4%** | Reached consensus | Reached consensus |
|  | How should the credentialling for each stage be decided? (tick all that apply) | | | |
| 26.1 | Minimum time allocation | 15.3% | 5.2% | n.a |
| 26.2 | Competency based (e.g. evidence of skills acquired) | **85.9%** | Reached consensus | Reached consensus |
| 26.3 | Proficiency based (e.g. mastery of skills acquired) | 58.8% | 57.1% | **85.2%** |
| 26.4 | Mastery level (above proficiency) | n.a | n.a | 17.3% |
| 27 | The training towards completing the credentialling process should be proficiency based (complete each part of training to benchmark) before progressing to the next stage of training | **84.7%** | Reached consensus | Reached consensus |
| 28 | Credentialling should involve benchmarking | **91.8%** | Reached consensus | Reached consensus |
| 29 | Do you agree that the benchmark for proficiency should be the median performance of a group of experts performing the same task or procedure? | 72.9% | **83.1%** | Reached consensus |
|  | How should we define the benchmarks? (tick all that apply) | | | |
| 30.1 | Avoidance of errors | 69.4% | 77.9% | **87.7%** |
| 30.2 | Autonomous activity | 55.3% | 37.7% | 35.8% |
| 30.3 | Time taken to complete task | 36.5% | 11.7% | 9.9% |
| 30.4 | Ability to operate at levels of complexity | 71.8% | 72.7% | 75.3% |
| 30.5 | Number of tasks completed | 60% | 28.6% | 37% |
| 30.6 | Ability to identify important anatomical structures | 70.6% | 71.4% | 76.5% |
| 30.7 | Awareness of set up and phases of the procedure | **82.4%** | Reached consensus | Reached consensus |
|  | How should procedures be assessed for credentialling? (Tick all that apply) | | | |
| 31.1 | Completion of modules within a key index procedure | **83.5%** | Reached consensus | Reached consensus |
| 31.2 | Task based completion | **84.7%** | Reached consensus | Reached consensus |
| 31.3 | Errors avoided | 58.8% | 35.1% | 53.1% |
| 31.4 | Identification of anatomy or phases of procedure | 67.1% | 67.5% | 71.6% |
| 32 | Credentialling should include a minimum number of cases observed | 61.2% | 64% | **87.7%** |
|  | What should this number be? | | | |
| 33.1 | n/a | 37% | n.a | n.a |
| 33.2 | 5-10 | 44% | **87%** | Reached consensus |
| 33.3 | 10-15 | 7% | 9.1% | n.a |
| 33.4 | 15 or above | 12% | 3.9% | n.a |
| 34 | Credentialling should include a minimum number of cases observed assisted? | 74.1% | **85.7%** | Reached consensus |
|  | What should this number be? | | | |
| 35.1 | n/a | 27% | n.a | n.a |
| 35.2 | 5-10 | 44% | **84.4%** | Reached consensus |
| 35.3 | 10-15 | 15% | 11.7% | n.a |
| 35.4 | >15 | 14% | 3.9% | n.a |
| 36 | Credentialling should include a minimum number of performed cases | **87.1%** | Reached consensus | Reached consensus |
|  | What should this number be? | | | |
| 37.1 | n/a | 14% | n.a | n.a |
| 37.2 | 5-10 | 31% | 36.4% | 27.9% |
| 37.3 | 10-15 | 21% | 15.6% | 21% |
| 37.4 | >15 | 35% | 48.1% | 51.9% |
| 38 | Should each specialty have a key index procedure in their credentialling process? | **92.9%** | Reached consensus | Reached consensus |
| 39 | Credentialling should involve use of video based assessment | 72.9% | **87%** | Reached consensus |
| 40 | Video based assessment should be used as part of final sign off | 68.2% | 79.2% | **91.4%** |
| 41 | There should be multiple videos submitted for assessment | 62.4% | 79.5% | **88.9%** |
| 42 | Submitted videos should be consecutive operations | 48.2% | 50.6% | 61.7% |
|  | How many consecutive videos should be submitted? | | | |
| 43.1 | Not consecutive | 50.6% | n.a | n.a |
| 43.2 | 2 | 5.9% | 10.4% | 8.6% |
| 43.3 | 3 | 27.1% | 71.4% | **85.2%** |
| 43.4 | 4 | 2.4% | n.a | n.a |
| 43.5 | 5+ | 14.1% | 18.2% | 6.2% |
| 44 | Video based assessment should be using key index procedures | 74.1% | **93.5%** | Reached consensus |
| 45 | There should be agreed, standardised video performance assessment metrics | 76.5% | **90.9%** | Reached consensus |
| 46 | Video based assessments should be benchmarked | 75.3% | **92.2%** | Reached consensus |
| 47 | Do you agree robotic training benefits from standardisation with agreed and defined performance metrics and benchmarks? | n.a | n.a | **97.5%** |
| 48 | Do you agree that for training related to a key index procedure, there needs to be agreement between the expert/trainer and trainee on what the performance metrics are. And that these metrics should be closely aligned with the experts approach? | n.a | n.a | **91.4%** |
| 49 | Given that many key index robotic procedures (such as Robotic-assisted radical prostatectomy) have different accepted approaches. Should we have 'Regional (local) Gold Standard' metrics (agreed between an appointed expert trainer/preceptor and their trainee) plus a 'Universal Gold Standard' for reference. With the Universal Gold standard approved by societies/organisations? | n.a | n.a | 66.7% |

|  | **Errors and metrics** | | | |
| --- | --- | --- | --- | --- |
| Item | Statement | Round 1 | Round 2 | Round 3 |
| 50 | Trainee operative metrics should be recorded | **89.4%** | Reached consensus | Reached consensus |
| 51 | Is it important to differentiate between consequential and non-consequential error (i.e the same technical error with or without consequence, e.g small bowel diathermy injury with or without enteric leak) | n.a | 79% | **92.6%** |
| 52 | Should we assess errors in credentialling assessment? | **83.5%** | Reached consensus | Reached consensus |
|  | How do we classify errors? | | | |
| 53.1 | Pre error e.g. dropping needle | 49.4% | 40.3% | 38.3% |
| 53.2 | Event error (e.g. losing needle) | 75.3% | 79.2% | **100%** |
| 53.3 | Non-consequential | 51.8% | 66.2% | 49.4% |
| 53.4 | Consequential | 88.2% | **92.2%** | Reached consensus |
| 54 | Robotic credentialling should include reassessment and revalidation | 70.6% | **80.5%** | Reached consensus |
|  | Revalidation should occur every: | | | |
| 55.1 | <5 years | 5.5% | 6.5% | 1.2% |
| 55.2 | 5 years | 79.5% | 77.9% | **87.7%** |
| 55.3 | 10 years | 15% | 15.6% | 11.1% |
| 56 | Credentialling should involve an audit of cases performed | **81.2%** | Reached consensus | Reached consensus |
| 57 | Robotic surgery should involve audit of all cases performed via a centralised registry | **82.4%** | Reached consensus | Reached consensus |

|  | **Credentialling access** | | | |
| --- | --- | --- | --- | --- |
| **Item** | **Statement** | **Round 1** | **Round 2** | **Round 3** |
| 58 | A trainee should be able to begin the credentialling process at any time (subject to access and availability) | **85.9%** | Reached consensus | Reached consensus |
| 59 | A trainee should be able to begin procedural aspects once the core aspects are completed (subject to access and availability) | **97.6%** | Reached consensus | Reached consensus |
|  | *When should you be eligible to begin the credentialling process (subject to access and availability)? (select all that apply)* | | | |
| 60.1 | Medical Student | 7.1% | 3.9% | 2.5% |
| 60.2 | Foundation training | 10.9% | 7.8% | 6.2% |
| 60.3 | Core training equivalent | 38.8% | 40.3% | 42% |
| 60.4 | Junior higher specialty training (ST3-5) | 64.7% | **83.1%** | Reached consensus |
| 60.5 | Senior higher specialty training (ST6-ST8) | 54.1% | 54.5% | **84%** |
| 60.6 | Post CCT | 42.4% | 41.6% | 45.7% |
| 61 | Robotic credentialling should be considered at ARCP | 77.6% | **90.9%** | Reached consensus |
| 62 | Credentialling should be independent of the completion of clinical training (CCT)? | **94.1%** | Reached consensus | Reached consensus |

**Supplementary table 2: Delphi consensus participants completing all 3 rounds**

| Michael El Boghdady | Ahmed Ghanem | William Maynard |
| --- | --- | --- |
| Conor Toale | Katerina Gkorilla | Azelle Egbe |
| Omar El Taji | Richard Mak | Thomas Kidd |
| James Walmsley | Vasudev Zaver | Walid Osama |
| William Atkins | Rajkiran Deshpande | Angela Lam |
| Sinead Ramjit | George Kourounis | Hannah Copley |
| Wee Juin Low | Taner Shakir | Usama Ahmed |
| Soham Bandyopadhyay | Emma Howie | Dominic Waugh |
| Oliver Pumphrey | Alex L Wojtowicz | Lara Manley |
| George Whittaker | Christopher Onyekachukwu Nwatuzor | Amanda Holden |
| Raiyyan Aftab | Elizabeth O'Connell | Srisha Duggineni |
| Setthasorn Ooi | Daniel Ashmore | Fabio Massimo |
| Rachel Coulson | Michael Okocha | Sally Rankin |
| David Robinson | Michal Kawka | Karen Chui |
| Raefe Jackson | Karen Dumont | Ross Warner |
| Gerard McKnight | Sajeed Ali | Fazia Hashim |
| Viraj Shah | Hatem Sadik | Kirsty Mozolowski |
| Matthew Harris | Marita Georgiou | Kala Kumaresan |
| Nathan Walker | George Davies | Aqua Asif |
| Benjamin Chan | James Archer | Emily Mills |
| Leo Brown | Jared Wohlgemut | Tiffany Li |
| Yan Mei Goh | Fraser Cullen | Helen Skinner |
| Joanna Aldoori | Fang Yi Cheung | Joe Mcloughlin |
| Jonathan Hirniak | Josh Burke | Andrew Yiu |
| Valdone Kolaityte | Kate Atkinson | Aidan Bannon |
| Mark Yao | Mudit Matanhelia |  |

**Supplementary table 3: Participating surgical trainee organisations**

| **Specialty organisation** | **Specialty** |
| --- | --- |
| Association of Surgeons in Training | Pan-specialty |
| Dukes Club | Colorectal surgery |
| Roux Group | Upper GI and Hepatobilliary surgery |
| Herrick Society | Transplant surgery |
| Trainees in Paediatric Surgery (TriPS) | Paediatric Surgery |
| Moynihan Academy | Emergency General Surgery |
| ALSGBI Academy | Minimally Invasive Surgery |
| Society of Cardiothoracic Surgery (SCTS) | Cardiothoracic Surgery |
| Roleaux club | Vascular Surgery |
| British Orthopaedic Trainee Association (BOTA) | Orthopaedic surgery |
| PLASTA | Plastic Surgery |
| British Neurosurgical Trainee Association (BNTA) | Neurosurgery |
| OMFS FiT | Maxillofacial surgery |
| Association of Otolaryngologists in Training (AOT) | ENT surgery |
| Mammary Fold | Breast Surgery |
| BAUS Section of Trainees (BSoT) | Urological surgery |
